# Novel Parvovirus in Pigs Associated with Exophthalmos and Erythema, the Netherlands

**DOI:** 10.3201/eid3208.251827

**Published:** 2026-08

**Authors:** Tijs J. Tobias, René Renzhammer, Charlotta A.J. Roos, Marrina G. Schuttert, Paul Waijers, Carmijn B. Meulenbroek, Karin Junker, José L. van Wilgen, Claudie Herbrink, Rick Elbert, Christiaan Sanderman, Eveline Willems, Remco Dijkman, Erhard van der Vries

**Affiliations:** Royal GD, Deventer, the Netherlands (T.J. Tobias, R. Renzhammer, C.A.J. Roos, K. Junker, J.L. van Wilgen, C. Herbrink, R. Elbert, C. Sanderman, E. Willems, R. Dijkman, E. van der Vries); Utrecht University, Utrecht, the Netherlands (T.J. Tobias); De Varkenspraktijk, Someren, the Netherlands (M.G. Schuttert, P. Waijers); De Oosthof Dierenartsen, Nijverdal, the Netherlands (C.B. Meulenbroek)

**Keywords:** viruses, parvovirus, swine, farms, eye, exophthalmos, strabismus, erythema, alopecia, Nanopore Sequencing, in situ hybridization, the Netherlands

## Abstract

We report an outbreak of infections with a novel protoparvovirus on several commercial farms in the Netherlands and associations with a clinical syndrome in pigs characterized by exophthalmos and erythema. Evidence of involvement of this virus, highly similar to a vulpine parvovirus, was substantiated by long-read sequencing and in situ hybridization techniques.

Parvoviruses are small, nonenveloped viruses with a single-stranded negative sense DNA genome (*1*). They are ubiquitous and infect a wide variety of hosts; disease manifestations range from subclinical to lethal (*1*). In pigs, 8 porcine parvoviruses (PPVs; PPV1–PPV8) are known; PPV1 is the species with established pathogenicity and is a major cause of reproductive disorders in pigs worldwide (*2*–*4*). Causal disease inference for other PPVs is challenging and requires a combination of virus detection and in situ hybridization (ISH), especially when histologic lesions are limited to nonexistent (*3*).

In December 2024, piglets (1–4 weeks of age) on 2 separate pig farms in the Netherlands displayed variable degrees of bilateral exophthalmos and strabismus and signs of erythema and alopecia (Figure 1). The cases were reported to Royal GD (Deventer, the Netherlands) as part of the national animal health surveillance program (*5*). All investigations on animals and animal tissues for this work were conducted under veterinary supervision for purpose of appropriate animal care and diagnostics, according to the Dutch Act on Animals, thereby exempt for ethical approval under the Dutch Experiments on Animals Act and EU Directive 2010/63/EU. Samples from control animals were obtained as part of another study for educational purposes at Utrecht University (animal welfare license number AVD10800202215910). The owners of the animals consented to all performed activities for this case report.

**Figure 1:**
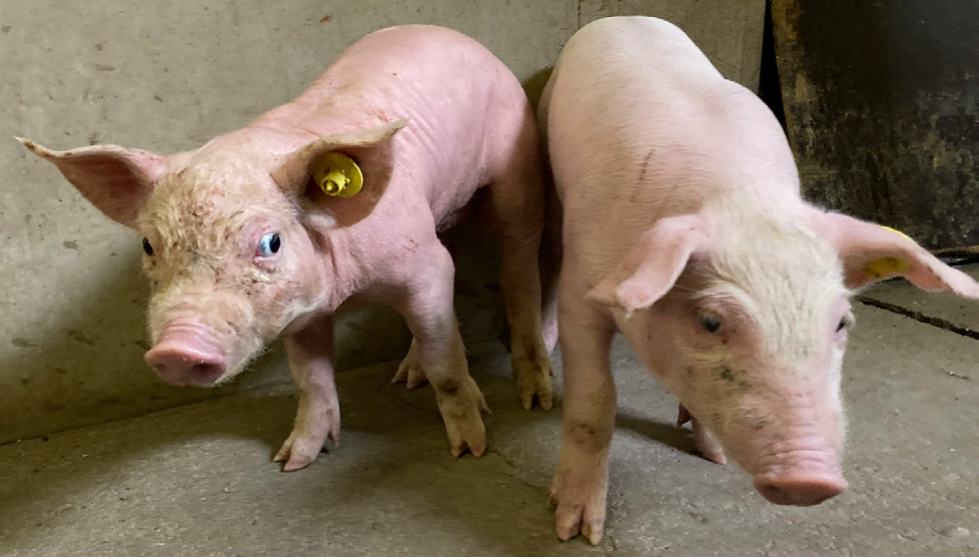
Case (left) and healthy control (right) piglets of ≈2.5 weeks of age in study of multifarm outbreak of novel parvovirus in pigs associated with exophthalmos and erythema, the Netherlands, 2024. Case piglet is shown to exhibit exophthalmos, strabismus, skin wrinkling, alopecia, and generalized erythema.

By August 2025, the number of farms reporting piglets showing those unusual clinical signs had increased to >80. Although the initial case farms indicated evidence of growth retardation, clinical findings had remarkably reduced after 3 to 4 months without applying specific interventions and without marked increase of death. Postmortem investigations were performed to study the underlying cause. Gross pathology of 20 piglets from 7 affected farms did not provide any conclusive results. Microscopic lesions were mostly absent or inconclusive. Finally, routine laboratory diagnostics for underlying nutritional or toxicological causes and molecular testing for common pig pathogens, including PPV1, were either negative or inconsistent. 

To identify a putative causative agent causing the unusual signs, we isolated nucleic acids from different tissues of 18 affected piglets and 4 nonaffected piglets (Appendix) and analyzed those nucleic acids by nontargeted Oxford Nanopore Technology (https://nanoporetech.com) sequencing and virus metagenomics using an in-house virus discovery pipeline (Appendix). We obtained near-complete parvovirus genomes (GenBank accession no. PX496757) from all affected piglets; the genome displayed >98% nucleotide identity to a species from the genus of Protoparvoviridae, which was previously detected in fecal samples from foxes (*6*,*7*). We confirmed the presence of viral DNA of that fox parvovirus in all affected piglets with clinical signs by a real-time PCR targeting the viral protein 2 region (Appendix); the lowest cycle threshold values were detected in liver and kidney tissue (range 21–34).

To further establish this virus as the causative agent, we applied ISH on several PCR-positive tissues (Appendix). We demonstrated presence of parvoviral nucleic acid by visualization of nucleic acid localized in the nuclei of kidney epithelial cells and hepatocytes (Figure 2, panel A), but PCR-positive eye and skin tissues remained ISH-negative. Finally, we attempted virus isolation using different cell lines and demonstrated virus replication by propagating the virus in swine testicular cells (Appendix) (*8*) without signs of cytopathologic effect (Figure 2, panel B).

**Figure 2:**
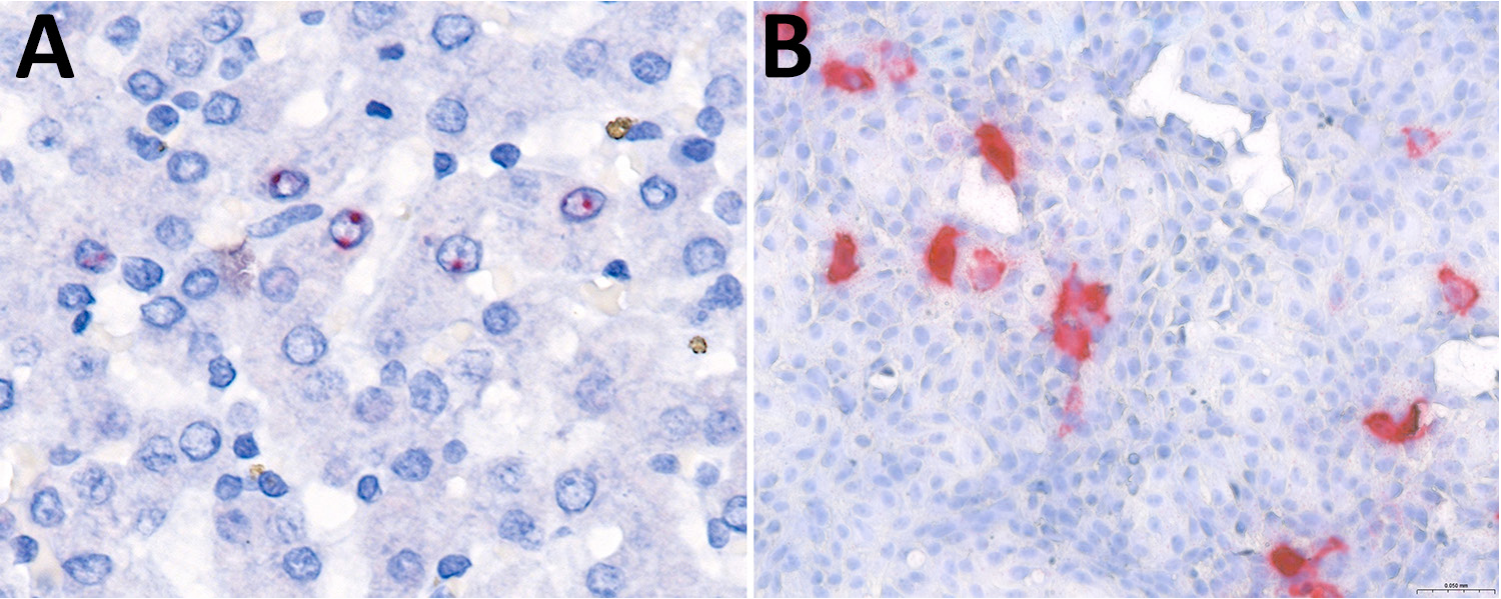
In situ hybridization from study of multifarm outbreak of novel parvovirus in pigs associated with exophthalmos and erythema, the Netherlands, 2024. A) Hepatocytes with red coloring depict Protoparvovirus nucleic acids, indicating focal presence of viral nucleic acids in nuclei of hepatocytes. In the center, viral DNA is present with 2 excentric foci in a cell nucleus, indicative of viral DNA presence in a cell in mitosis (brightfield scan of histology slide, 40× objective magnification [0,19 µm/pixel]). B) In situ hybridization of swine testicular cell line infected with protoparvovirus. Original magnification 10×10.

Our findings suggest that this novel parvovirus replicates efficiently in multiple organs of young piglets displaying exophthalmos and erythema. Still, absence of microscopic lesions in the affected pigs and the negative ISH results for ocular and dermal tissue suggest that the clinical signs are secondary to other pathophysiological processes, possibly because of unrevealed cardiovascular effects. Parvoviruses frequently require cofactors or co-infections to result in pathologic results and clinical signs (*1*). Whether erythema and alopecia are directly caused by parvovirus infection has yet to be investigated, but dermal pathology in pigs has been described during PPV1 infection (*9*).

Although the most recent ancestor to this novel virus is a virus previously detected in feces of foxes in the Netherlands in 2012 (*6*) and Croatia in 2016 (*7*), its origin could be from their diet (*1*,*6*). Recent information about fox parvovirus in wildlife in the Netherlands is not available, and how this virus was initially introduced in the domestic pig population in the Netherlands remains unclear. An apparent spillover to pigs was recently described for another protoparvovirus and was suggested to result from contact with wildlife feces (*10*).

Because the detected parvovirus is genetically different from PPV1 and all case and control farms apply routine vaccination for PPV1, cross immunity is expected to be low. Final proof of causality of this parvovirus to lead to clinical signs is needed. Follow-up studies should focus, among other topics, on the transmission routes and effects of the virus on production, swine health, and welfare. Veterinarians and pig farmers in this region should be aware of this novel virus and its potential manifestations. 

Appendix Additional information about multifarm outbreak of novel parvovirus in pigs associated with exophthalmos and erythema, the Netherlands, 2024.
