# Supplementary material for: Novel Parvovirus in Pigs Associated with Exophthalmos and Erythema, the Netherlands
Source: Emerg Infect Dis. 2026 Aug;32(8):1376–8. doi: 10.3201/eid3208.251827 (PMC13426867; doi:10.3201/eid3208.251827)
Supplement: Appendix — Additional information about multifarm outbreak of novel parvovirus in pigs associated with exophthalmos and erythema, the Netherlands, 2024. [file 25-1827-Techapp-s1.pdf]

Article DOI: https://doi.org/10.3201/eid3208.251827

EID cannot ensure accessibility for supplementary materials supplied by authors. Readers who have difficulty accessing supplementary content should contact the authors for assistance.

Novel Parvovirus in Pigs Associated with Exophthalmos and Erythema, the Netherlands

Appendix

1. Sample collection

Tissue (kidney, liver, brain, ocular, skin, myocard, intestine, lung) and serum samples were collected from clinically affected piglets as part of *post-mortem* investigations at Royal GD (Deventer, the Netherlands), as well as samples from non-affected control piglets allegedly from non-affected farms, investigated for educational purposes at and by Utrecht University and obtained with permission. Only from three submissions eye samples were available. From three other farms, kidney, liver, brain, myocard and/or intestinal tissue was analyzed leading to 30 samples in total of seven case farms.

2. Nucleic acid extraction and amplification

Nucleic acids were isolated from samples using the MagMAX Pathogen RNA/DNA extraction method on a KingFisher Flex system. In brief, 150 mg of sample was added to a BioSpec bead tube containing 1.5 mL PBS and homogenized in the FastPrep-24 system. Suspensions were subsequently filtered using a 0.8 μM PES filter. The filtrate was treated using benzonase and micrococcal nuclease before NA extraction. RNA/DNA was eluted in 75μl elution buffer. Enrichment of viral nucleic acid was performed using the NetoVIR SeqPlex amplification kit (Sigma, SEQR-50RXN).

3. ONT sequencing

Library preparation was carried out using the Oxford Nanopore Technologies (ONT) Native Barcoding Kit 24 V14 (SQK-NBD114.24). Sequencing was conducted on a GridION system (ONT), with a runtime of 48 hours and basecalling in the 'Super-accurate' mode. Adaptor

trimming and quality control were performed with the following parameters: trim using a quality score = 0.05 quality limit, trim 5′ ends = 25 nt, trim 3′ ends = 25 nt, discard short reads <200 nt.

4. Metagenomics

Host depletion against the swine genome (*Sus scrofa* (Sscrofa11.1)) was performed on the trimmed reads. Unmapped reads were mapped against the microbial, UNITE, LSU, SSU, and viral databases using the Taxonomic profiling tool (v2.38) of the CLC Microbial Genomics module (24.1.1) with a minimum seed length of 30. Subsequently, reads were mapped against the best matching viral reference from the taxonomic profiling using minimap2 in CLC Genomics Workbench (24.0.2) and reference KC692368. Whole genome alignment was performed using MUSCLE alignment with full genome consensus sequences obtained from the nine virus discovery samples and of 51 reference strains from nine parvovirus genera retrieved from Genbank.

5. Novel parvovirus real-time PCR assay

Real-time PCR was performed with two different PCR's targeting two regions (nucleotides 2853–3026 and nucleotides 4204–4416) in the VP2 of the fox parvovirus genome [Genbank accession number KC692368]). Primer sequences were as follows: for PCR1, forward primer 5′-AGCCCATTCAACCAGACTCG-3′, reverse primer 5′-CATGCGTTGCAATCCACCAA-3′ and probe 56-FAM/CACTGCTGG/ZEN/TGCTGC TGGGA/3IABkFQ; for PCR2, forward primer 5′- TCACCAACCAGTGCAACCTT-3′, reverse primer 5′- CTTTCTTGGTTTCCAGCCGC-3′ and probe 56-FAM/CCACAATGG/ZEN/AGCCTGGGAGCA /3IABkFQ. The PCR was performed on the Quantstudio 5 (Applied Biosystems, Life Technologies, CA, USA) using the AgPath-ID one-step RT PCR kit (Applied Biosystems) and ROX dye for normalization. The baseline correction was performed on cycles 3–15. The threshold for the FAM detector for PCR 1 was set at 0.2 and for PCR 2 at 0.15. Samples with a Ct value above 40 are considered negative. The PCR program was as follows: initial denaturation/enzyme inactivation, 10 minutes at 95°C; 45 cycles of 15 seconds at 95°C and 45 seconds at 60°C.

6. In situ hybridization

In situ hybridization was performed on sections from formalin fixed paraffin embedded tissues, using RNAscope® 2.5 HD Detection Reagent-RED system [Advanced Cell Diagnostics (ACD), REF 322360], in combination with a RNAscope probe.

Slides were baked for 1 h at 60°C. Deparaffinization was performed by 2x 5 min in xylene and 2x 1 min of 100% ethanol. Pre-treatment of the FFPE slides included 10 min at room temperature with RNAscope® hydrogen peroxide [Advanced Cell Diagnostics (ACD), REF 322381]. Antigen Retrieval was done by cooking for 10 minutes in 1X Envision FlexTarget Retrieval Solution (pH 9,0), [Agilent Dako, art.nr. K8000], and enzyme pretreatment for 10 min at RT with RNAscope® Protease Plus [Advanced Cell Diagnostics (ACD), REF 322381].

The following steps were completed according to the RNAscope® Assay using the RNAscope® 2.5 HD Detection Reagent—RED. The slides were incubated for 2 h in the HybEZ Oven at 40°C for 2 h with RNAscope probe. The amplification steps were performed as described in the user manual. Signal was detected with Fast RED with a 10-min room temperature incubation. The slides were counterstained with 50% Gill's hematoxylin I and mounted with EcoMount.

7. Virus isolation

Swine testicular (ST) 1 cells were grown in DMEM (GIBCO, ref:41965062) supplemented with 10% fetal calf serum (FCS). Tissues were homogenized and 150 mg of organ material was added to a Biospec Bead tube containing 1mm Zirconia beads (BiosSpec product inc, ref: 11079110) and 1.5ml growth medium. Three homogenization cycles were performed with the FastPrep-24 system at 5.5 m/s for 10 seconds with a 30 second cooling interval. The homogenates were centrifugated at 13,000 x *g* for 5 minutes, after which the supernatant was collected. Prior to infection, the growth medium was removed from the Falcon® T25 flasks (Corning, ref: 353082) containing a 50% confluent ST1 monolayer. One mL of the collected supernatant was used for the infection of the ST1 cells and incubated for 1 hour at 37°C, 5% CO_2. After the absorption, the cells were washed thrice with PBS pH 7.2 (GIBCO, ref: 20012068). Growth medium supplemented with antibiotics (GIBCO, ref: 152400620) was subsequently added to each flask. The infected ST1 cells were incubated at 37°C, 5% CO_2 for 5 days. 0.25ml of the medium was harvested at 2 and 5 days post infection for DNA/RNA

isolation, as previously described, for detection of novel parvo virus with the real time PCR assay.
